# Fecal pellets of giant clams as a route for transporting Symbiodiniaceae to corals

**DOI:** 10.1371/journal.pone.0243087

**Published:** 2020-12-16

**Authors:** Masami Umeki, Hiroshi Yamashita, Go Suzuki, Taiki Sato, Shizuka Ohara, Kazuhiko Koike

**Affiliations:** 1 Graduate School of Integrated Sciences for Life, Hiroshima University, Kagamiyama, Higashi-Hiroshima, Hiroshima, Japan; 2 Fisheries Technology Institute, Japan Fisheries Research and Education Agency, Okinawa, Japan; Evergreen State College, UNITED STATES

## Abstract

Because more than 80% of species of gamete-spawning corals, including most Acroporidae species, do not inherit Symbiodiniaceae from their parents, they must acquire symbiont cells from sources in their environment. To determine whether photosynthetically competent Symbiodiniaceae expelled as fecal pellets from giant clams are capable of colonizing corals, we conducted laboratory experiments in which planula larvae of *Acropora tenuis* were inoculated with the cells in fecal pellets obtained from *Tridacna crocea*. *T*. *crocea* fecal pellets were administered once a day, and three days later, cells of Symbiodiniaceae from the fecal pellets had been taken up by the coral larvae. *T*. *crocea* fecal pellets were not supplied from the 4^th^ day until the 8^th^ day, and the cell densities in the larvae increased until the 8^th^ day, which indicated the successful colonization by Symbiodiniaceae. The control group exhibited the highest mean percentage of larvae (100%) that were successfully colonized by culture strains of Symbiodiniaceae, and larvae inoculated with fecal pellets reached a colonization percentage of 66.7 ~ 96.7% on the 8^th^ day. The highest colonization rate was achieved with the fecal pellets containing cells with high photosynthetic competency (*Fv/Fm*). Interestingly, the genetic composition of Symbiodiniaceae in the larvae retrieved on the 8^th^ day differed from that in the fecal pellets and showed exclusive domination of the genus *Symbiodinium*. A minor but significant population of the genus *Cladocopium* in the fecal pellets was not inherited by the larvae. These experiments provided the first demonstration that the Symbiodiniaceae from tridacnine clams provided via fecal pellets can colonize and even proliferate in coral larvae.

## Introduction

Coral reefs are habitats with high biodiversity in which approximately one-quarter to one-third of all marine species are found, while coral reefs cover only 0.2% of the ocean’s surface [1–3]. Reefs are fundamentally built by stony corals, which mainly rely on nutrition from symbiotic algae, and these algae are often called zooxanthella(e). The word zooxanthella is broadly used to indicate a group of golden-brown microalgae engaging in symbiosis with various host animals [4], but a specific group of dinoflagellates in the Symbiodiniaceae family, which are traditionally referred to as genetic clades A to I [5] and were recently formally described as genera [6], has more commonly been treated as the representative group of zooxanthella.

Symbiodiniaceae are essential for host species. Contrary to the majority of brooding corals that maternally inherit symbiont cells (vertical transmission), more than 80% species of gamete-spawning corals must acquire their own symbionts from their surrounding environments (horizontal transmission) [7]. Hence, environmental pools of the Symbiodiniaceae population could play elemental roles to supply symbionts for juveniles and/or adults of these host animals [8]. Consistent with this view, several studies have addressed the genetic diversity of Symbiodiniaceae in the environment [8–14], their abundances [13, 15] and the similarity or dissimilarity between coral juveniles and host-free populations in sediments [16]. Corals discharge their symbiont cells into the surrounding environments [17–21], and these cells can then accumulate in the environment [13]. Thus, these discharged cells from coral hosts might be a candidate symbiont source for other corals. Some cnidarian hosts expel Symbiodiniaceae that undergo cell division [22] or maintain competency in the photosystem [17, 18], and a previous study showed that significant percentage of the discharged cells from a coral are degraded or damaged [21].

Another component of the coral reef ecosystem, tridacnine clams (giant clams), which bear Symbiodiniaceae symbionts in their flesh bodies, also discharge these cells in their fecal pellets [23–26]. In contrast to corals, Symbiodiniaceae reside in “zooxanthellal tubes”, which elongate from the giant clam’s stomach and spread into the mantle area [27]. Although it appears that the majority of cells underwent digestion, 1.6–11.8% of the zooxanthellae populations in a giant clam could be expelled in intact form [24, 26] and photosynthetically active [23, 26]. Morishima et al. [26] first demonstrated that overflowed and expelled Symbiodiniaceae, such as those in the fecal pellets of *Tridacna crocea*, are capable of colonizing *Tridacna squamosa* larvae. They further suggested the possibility that fecal pellets could serve as vectors of symbionts to other animals (e.g., corals), and this hypothesis should be further investigated.

In this study, we conducted laboratory experiments to demonstrate whether Symbiodiniaceae in *T*. *crocea* fecal pellets colonize in aposymbiotic coral juveniles. Fecal pellets obtained from *T*. *crocea* were supplied to planula larvae of *Acropora tenuis* once a day for three continuous days, and the larvae were further maintained without fecal pellets for five days to confirm stability of the colonization of the symbiont cells in the larvae.

## Materials and methods

### Preparation of coral larvae

Three parental *Acropora tenuis* were collected from around Ishigaki Island, Okinawa, Japan, on 8^th^ May 2019 (Permission No. 31–1 issued by the Okinawa Prefectural Government for research use). They were separately placed in a pail, and spawning was artificially induced with the addition of H_2_O_2_ [28]. Once the egg-sperm bundles were released from all these corals, they floated on the surface of the pail water. The bundles were then collected with a pipette and mixed in filtered seawater in a 5-L container, which resulted in cross-fertilization among the different coral colonies. The fertilized eggs were washed with 0.2-μm-filtered seawater (FSW) to remove the remaining sperm and any unexpected symbiont contamination. Until the experiment, approximately 1000 larvae were maintained in two 1-L polycarbonate containers that were placed in an incubator at 27°C with a light regime of 30 μmol photon m^-2^ sec^-1^ (12-h light/12-h dark period; SPECTRA SP200, Blue Harbor, Osaka, Japan). The seawater was changed daily. Planula larvae on the 3rd day after fertilization were randomly selected from the containers for use in the following inoculation tests.

### Preparation of fecal pellets of giant clams

**Fig 1:**
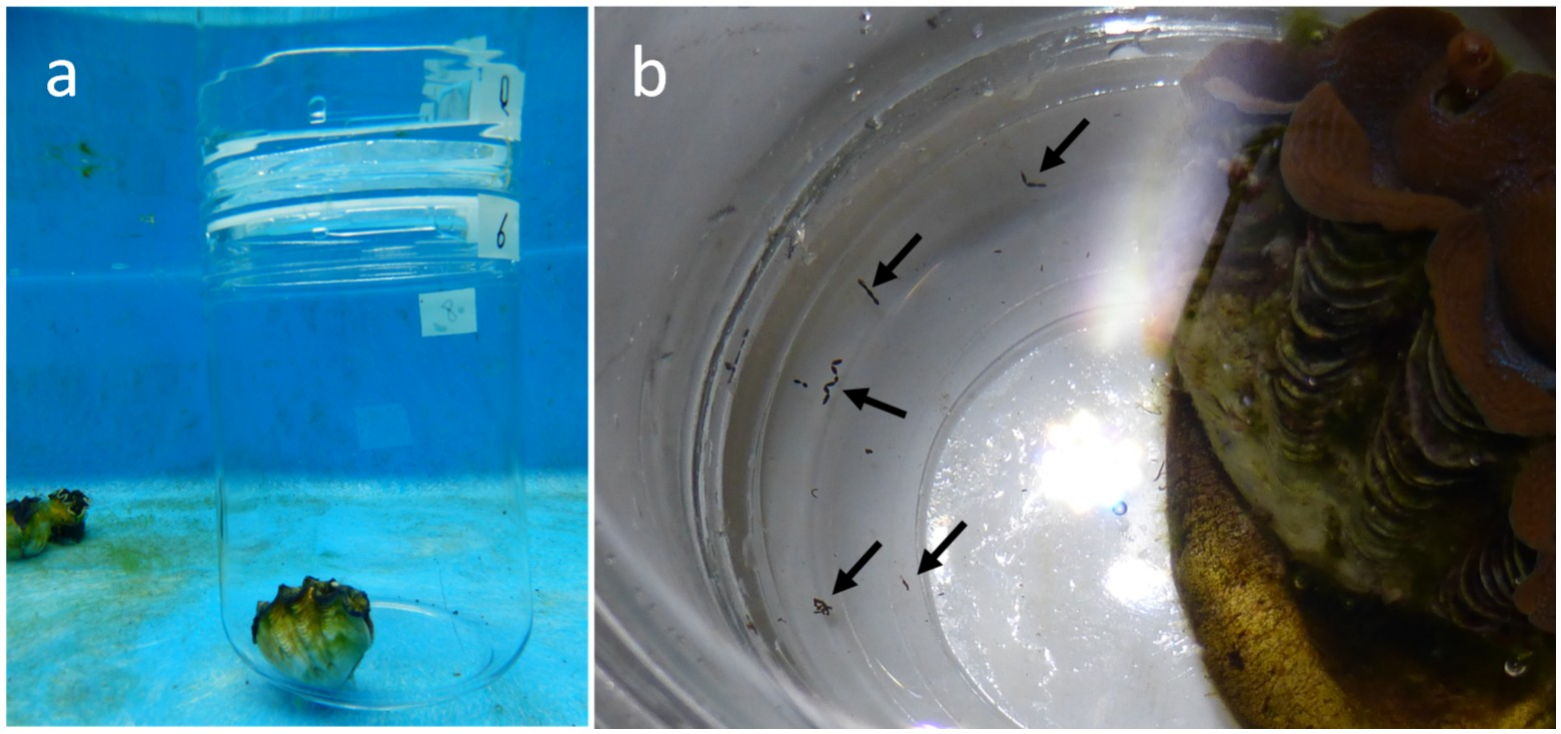
Incubation of *Tridacna crocea* in a jar for the collection of fecal pellets. (a) Incubation of a jar submerged in an aquarium tank. (b) Expelled fecal pellets at the bottom of the jar (arrows).

Three individuals of *T*. *crocea* were collected from Urasoko Bay (24°28’ N, 124°13’ E), Ishigaki, Okinawa, Japan, on May 2019 (Permission No. 30–82 issued by the Okinawa Prefectural Government for research use) and were maintained in an outdoor running seawater tank for 4 days. The shell lengths were 70, 55 and 48 mm for individuals 1, 2 and 3, respectively. To collect fecal pellets, each of the individuals was separately placed in a 0.8 L glass jar (113 mm φ × 168 mm H) filled with FSW, and the jars were again submerged in the tank to avoid an increase in temperature. After 5 h of incubation (9 am–2 pm), the giant clams were removed, and 20–30 of the fecal pellets that accumulated at the bottom of the jar were retrieved with a pipette (Fig 1). The pellets were gathered in a Petri dish containing FSW and transferred to a 1.5 mL microtube. The pellets were washed several times in the tube with FSW and lightly homogenized using a hand pestle until the pellets crumbled. A portion of the homogenate was examined with a fluorescence microscope under blue light excitation (BX50; Olympus, Tokyo, Japan), which revealed that numerous symbiont cells with bright chlorophyll *a* fluorescence were present in the pellets. To determine their photosynthetic competency, the maximum quantum yields of photosystem II (*Fv/Fm*) of those cells in the homogenate were measured using a microscopy-type pulse amplitude-modulated (PAM) fluorometer (Micro-FluorCam FC 2000, Photon Systems Instruments, Brno, Czech Republic) at the single-cell level, following the protocol of Fujise et al. [21]. The measurements were performed separately for all three giant clam individuals. A total of 26 ~ 129 cells were subjected to this measurement. Another portion of the homogenates was collected and diluted with FSW, and the cell density mL^-1^ was determined under a microscope. These homogenates were further supplied to the coral larvae. The fecal pellets obtained from the individual 1, 2 and 3 are hereafter abbreviated FP1, FP2 and FP3, respectively. The remaining homogenates in the microtube were stored in a freezer to determine the genetic composition of Symbiodiniaceae.

### Culture of Symbiodiniaceae strains

Four culture strains were used as controls in the experiment: AJIS2-C2 (former clade A type A1; *Symbiodinium microadriaticum*), CCMP2556 (former clade D type D1-4; *Durusdinium trenchii*), TsIs-H4 (former clade A type A6; *S*. *tridacnidorum*) and TsIs-G10 (former clade D type D4-5; *Durusdinium* sp.). CCMP2556 was purchased from the Provasoli–Guillard National Center for Culture of Marine Algae and Microbiota (ME, USA). Other strains were our originals. Among them, AJIS2-C2 (*S*. *microadriaticum*) and CCMP2556 (*D*. *trenchii*) are known to be well acquired by *A*. *tenuis* larvae under laboratory conditions [29]. TsIs-H4 and TsIs-G10 were originally isolated from *Tridacna squamosa* mariculture seeds at the Okinawa Prefectural Fisheries Research and Extension Center. All the cultures were maintained in an incubator at 27°C under a light regime of 40–50 μmol photon m^-2^ sec^-1^ (12-h light/12-h dark period; SPECTRA SP200) in IMK medium (Wako Jyunyaku, Tokyo, Japan). Prior to subjecting them to the experiment, the *Fv*/*Fm* of a total of 9 ~ 60 cells was measured using the microscopy-type PAM fluorometer, following the protocol mentioned above.

### Inoculation experiment

A total of 18 tubes (plastic 50 mL centrifuge tubes, 430829, Corning, NY, USA) were prepared for the overall experimental groups. Three of the groups (triplicated = 9 tubes) were provided fecal pellets from the three different *T*. *crocea* individuals. The other two groups (triplicated = 6 tubes) were used as controls and treated with a mixture of the Symbiodiniaceae culture strains. The remaining three tubes served as negative controls and were not provided any symbiont sources. Fifty 3-day-old larvae were placed into each tube filled with FSW. For the groups provided with fecal pellets, a portion of the abovementioned diluted homogenate of the fecal pellets (equivalent to 1,500 cells of Symbiodiniaceae) was added to each tube, resulting in a cell density of 30 cells mL^-1^. Three tubes were prepared for the fecal pellets obtained from a *T*. *crocea* individual. The same treatment was separately applied using the fecal pellets obtained from the two other *T*. *crocea* individuals. As controls, mixtures of culture strains of Symbiodiniaceae were added to the remaining six tubes; three tubes contained the same cell numbers (750 cells) of AJIS2-C2 and CCMP2556 (hereafter denoted C1), and the three other batches contained TsIs-H4 and TsIs-G10 (hereafter denoted C2). No symbiont cells were added to the remaining three tubes as negative controls. These tubes were placed on a diagonal rotator in the above mentioned incubator, and the rotation speed was set to 0.25 rpm (S1 Fig). During the first 3 days, fecal pellets were obtained daily and supplied once a day. Mixtures of the culture strains were also supplied to the control tubes once a day. From the 4th day until the end of the incubation period (8^th^ day), no symbiont sources were added (see S2 Fig for the experimental scheme). The filtered seawater in the tubes was changed daily during the first 3 days prior to the addition of the symbiont sources and on the 4^th^ and 6^th^ days.

### Observation of larvae

Because repeated sampling might cause unwanted damage to the coral larvae, observations were twice, on the 3rd day and 8th day of the inoculation experiment. On the 3rd day, ten larvae were retrieved directly from each tube with a pipette, and a total of 30 larvae per treatment (i.e., a type of symbiont source) were observed with a fluorescence microscope under violet light excitation (BX50; Olympus) to determine the percentages of the larvae that were colonized with symbiont cells and the cell density per larva. These observations were also made on the 8^th^ day. Therefore, a total of 30 larvae were assumed to remain in a tube. Because the discrimination of living versus dead individuals was difficult even in the absence of motility, the survival rates at the end of the experiment could not be determined. However, at the end of the experiment, 25–30 individuals remained in a tube, and most of these larvae (roughly more than 20 individuals) kept swimming. These larvae were transferred by a pipette to 1.5 ml tubes and kept in a freezer for further identification of Symbiodiniaceae genera.

### Genetic compositions of Symbiodiniaceae within the fecal pellets and the colonized larvae

The frozen fecal pellets from each giant clam source were obtained daily until the 3^rd^ day, thawed at room temperature, and added with 500 μl of TE buffer (pH 8.0). Total DNA was extracted by the TE-boiling method [30]. The frozen larvae obtained on the 8^th^ day were also thawed at room temperature and subjected to the phenol/chloroform method using CHAOS solution (4 M guanidine thiocyanate, 0.1% N-lauroyl sarcosine sodium, 10 mM Tris pH 8.0, 0.1 M 2-mercaptoethanol) following the protocol in [31]. Giant clams usually harbor Symbiodiniaceae, *Symbiodinium*, *Cladocopium*, and/or *Durusdinium* in their bodies [i.e., 32]; thus, the cell numbers of these genera in each extracted DNA sample were quantified by quantitative PCR (qPCR) based on the system described in Yamashita et al. [20].

### Cloning and sequencing of Symbiodiniaceae in fecal pellets and larvae

To identify finer taxonomic levels (i.e., species or types) contained in the fecal pellets and the colonized larvae, DNA cloning was performed. Among the three treatments incubated with fecal pellets from the different individuals of *T*. *crocea*, one treatment (fecal pellets obtained from the individual 3; FP3) showing the highest density of colonized cells was selected. The colonized larvae retrieved on the 8^th^ day (from each of the triplicate tubes; total of three samples) and the fecal pellets collected daily until the 3^rd^ day were used for this identification. Although we did not count the number of larval individuals, we assumed that approximately 90 individuals were included in this analysis based on an assumption that 30 of the initial 50 individuals (10 sacrificed on the 3^rd^ day and 10 sacrificed on the 8^th^ day) remained in each of the three tubes. The fecal pellets collected daily over three days were combined for the analysis. Internal transcribed spacers 1 and 2 (ITS-1, -2) and the whole region of the nuclear 5.8S rRNA gene were PCR-amplified with a PCR primer set of r18Sf and Sym28Sr [33]. The PCR products were cloned using a pCR4-TOPO TA cloning kit (Invitrogen, Carlsbad, CA, USA) and sequenced. Totals of 116 readings for the larvae and 49 readings for the stored fecal pellets were obtained. They were classified into species according to GeoSymbio [34] and/or through a query via International Nucleotide Sequence Database (INSD).

### Statistical analysis

The statistical tests were performed using R version 3.6.3 [35]. We performed a likelihood ratio test based on a generalized linear model with mixed effects (GLMM) to examine whether the Symbiodiniaceae cell numbers within colonized larvae significantly changed between the two observation days (3^rd^ day vs. 8^th^ day). For this modeling, we used the package glmmTMB version 1.0.1 [36]. The Symbiodiniaceae cell numbers within individual colonized larvae were assumed to follow a negative binomial distribution with a quadratic parameterization, and the link function was log [37]. We also applied poisson distribution and negative binomial distribution with linear parameterization, for our data; however, the smallest Akaike information criterion (AIC) was recorded when negative binomial distribution: quadratic parameterization was applied to the data. The explanatory variable was observation days. The random effect was assumed to vary among the experimental tubes. The likelihood ratio test was utilized to comnpare this model and the null model excluding explanatory variables (observation days) using the “anova” function in R. We separately conducted these analyses for each symbiont source. One-way analysis of variance (ANOVA) was used to determine the differences among the *Fv/Fm* values of the five symbiont source groups excluding the negative control. The cellular *Fv/Fm* values of each Symbiodiniaceae source inoculated during the first 3 days in the inoculation experiment (1^st^ day, 2^nd^ day and 3^rd^ day) were combined and analyzed: FP1 consisted of a total of 165 cells (65, 50 and 50 cells on the 1^st^ day, 2^nd^ day and 3^rd^ day, respectively), FP2 consisted of 217 cells (52, 129 and 36 cells), FP3 comprised 175 cells (110, 26 and 39 cells), the culture strain mixture of C1 consisted of 102 cells (33, 9 and 60 cells), and C2 comprised 78 cells (27, 22 and 29 cells). The null hypothesis was that there were no differences among the five symbiont sources. If the null hypothesis was rejected, we subsequently performed the post-hoc Tukey HSD test for multiple comparisons. In the present study, *p*-values less than 0.05 were considered statistically significant.

## Results

### Photosynthetic competency of the symbiont sources

**Fig 2:**
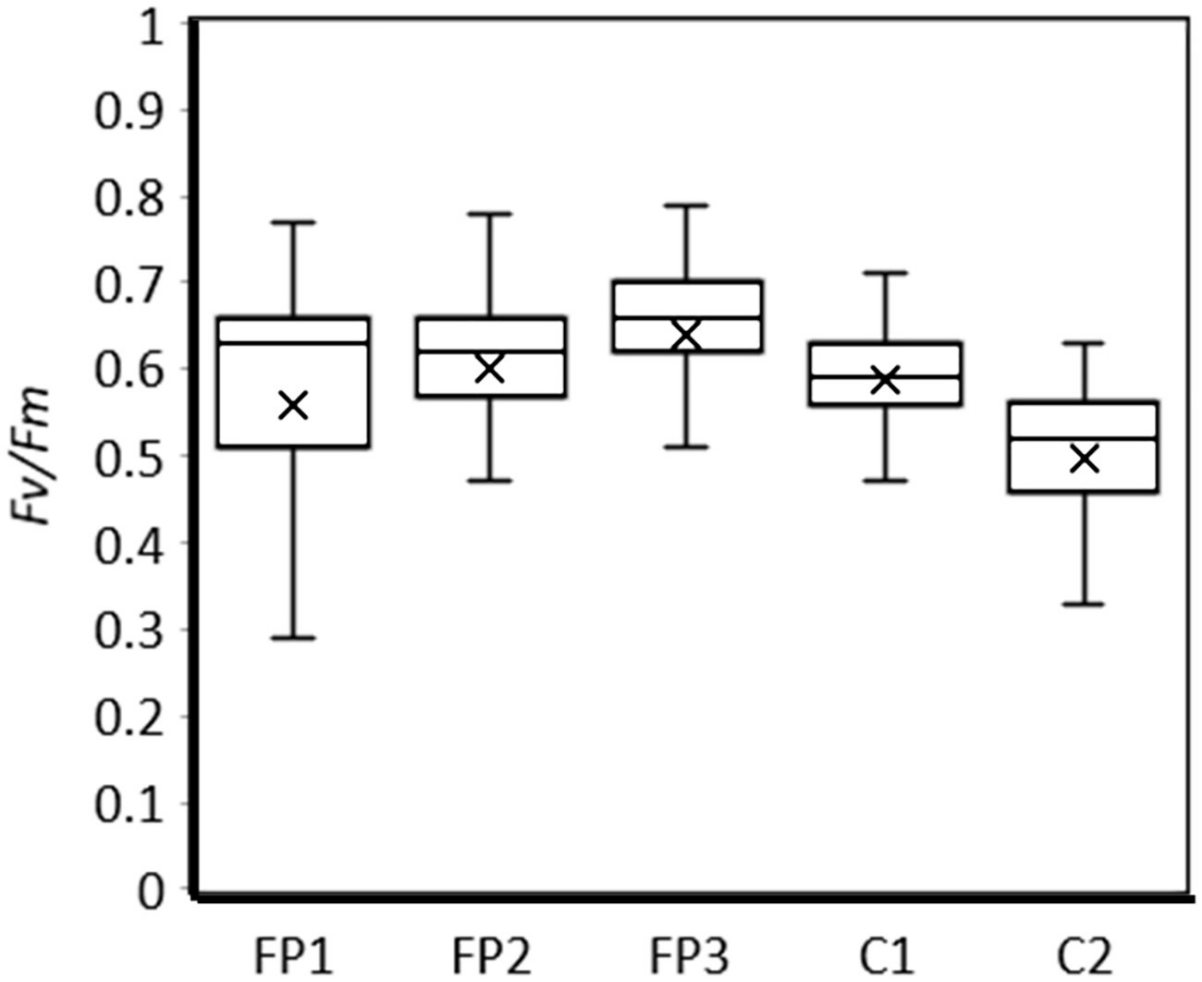
Box plot of *Fv/Fm* values of the symbiont cells in the fecal pellets of *Tridacna crocea* and the culture strains. FP1, FP2 and FP3 are the fecal pellets from different individuals of *Tridacna crocea*. C1 refers to a mixture of AJIS2-C2 (*Symbiodinium microadriaticum*) and CCMP2556 (*Durusdinium trenchii*), and C2 is a mixture of TsIs-H4 (*S*. *tridacnidorum*) and TsIs-G10 (*Durusdinium* sp.). The boxes show the quartiles with maximum and minimum values as the vertical bars and the medians as the horizontal bars. The averages are shown as x.

The *Fv/Fm* values of the cells in FP1, FP2, FP3, C1 and C2 are shown in Fig 2. Throughout the inoculation period (total of 3 days), the average *Fv/Fm* values for the symbiont sources FP1, FP2, FP3, C1 and C2 were 0.56 ± 0.17, 0.60 ± 0.14, 0.64 ± 0.13, 0.59 ± 0.06 and 0.50 ± 0.10, respectively (average ± SD). In particular, the *Fv/Fm* values of FP3 were significantly higher than those of the other pellets (*p*< 0.05, Tukey HSD).

### Inoculation coral larvae with the symbiont cells

**Fig 3:**
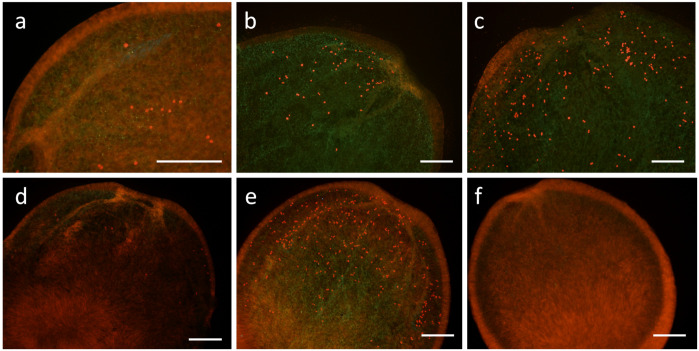
Fluorescent micrographs of *Acropora tenuis* larvae on the 8^th^ day showing the acquisition of symbiont cells (bright red fluoresced dots). (a)~(c) Larvae inoculated with fecal pellets of different individuals of *Tridacna crocea*. (d) A larva provided the culture strains of AJIS2-C2 (*Symbiodinium microadriaticum*) and CCMP2556 (*Durusdinium trenchii*). (e) A larva provided TsIs-H4 (*S*. *tridacnidorum*) and TsIs-G10 (*Durusdinium* sp.). (f) A larva that was not provided the fecal pellets or culture strains. Scale bars = 200 μm.

On the 3^rd^ day, symbiont cells were found only in the larvae supplied with the symbiont sources, whereas no recognizable cells were found in the larvae that were not given a symbiont source. The symbiont cells were distributed around the larval mouth. Both the larvae supplied the fecal pellets (Fig 3a–3c) and those given the culture strains (Fig 3d and 3e) took up the symbiont cells, and the cell numbers were higher on the 8^th^ day.

**Fig 4:**
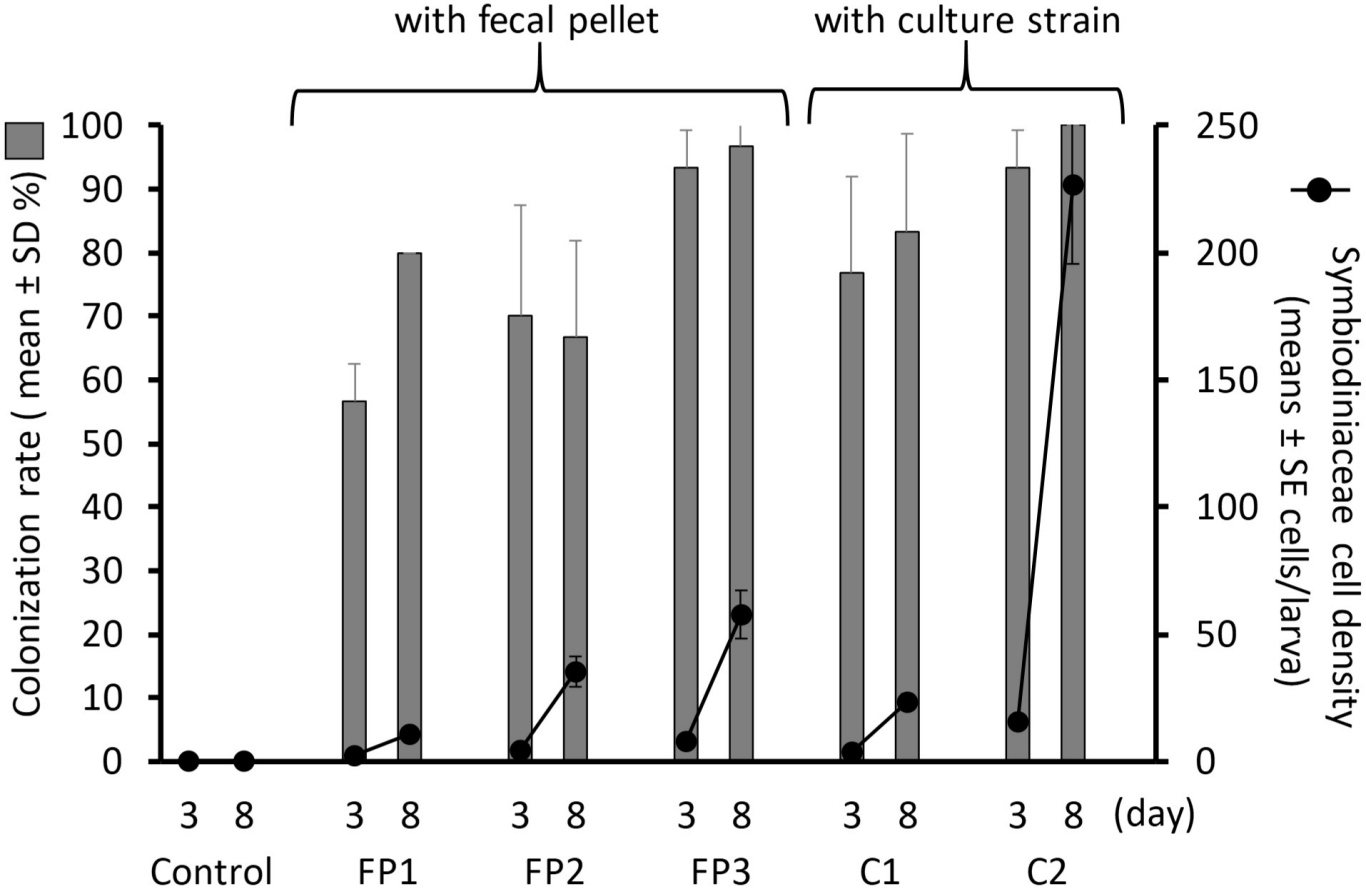
Proportions of *Acropora tenuis* larvae colonized with symbiont cells (bars) and colonizing cell densities per larva (lines with dots). FP1, FP2 and FP3 represent the experimental groups larvae provided fecal pellets from different individuals of *Tridacna crocea*. C1 and C2 refer larvae supplied with a mixture of AJIS2-C2 (*Symbiodinium microadriaticum*) and CCMP2556 (*Durusdinium trenchii*) and a mixture of TsIs-H4 (*S*. *tridacnidorum*) and TsIs-G10 (*Durusdinium* sp.), respectively. The control group was not provided any symbiont source.

On the 3rd day, the colonization rates, i.e., the mean proportions (over the triplicated tubes) of successfully colonized larvae, obtained for the experimental groups FP1, FP2, FP3, C1, and C2 and the control group, were 56.7 ± 5.8%, 70.0 ± 17.3%, 93.3 ± 5.8%, 76. 7 ± 15.3%, 93.3 ± 5.8% and 0% (means ± SDs from triplicate experiments), respectively (Fig 4, bars). On the 8th day, the colonization rates were slightly increased in most experimental groups, and those found for the FP1, FP2, FP3, C1, C2 and the control groups reached 80.0 ± 0.0%, 66.7 ± 15.3%, 96.7 ± 5.7%, 88.4 ± 15.3%, 100 ± 0.0% and 0% (means ± SDs from triplicate experiments), respectively. Among the experimental groups provided fecal pellets, the highest colonization rate was found for the FP3 group on the 8^th^ day. The mean cell densities per larva obtained for the FP1, FP2, FP3, C1 and C2 groups on the 3^rd^ day were 2.53 ± 0.44, 4.52 ±0.78, 7.79 ± 1.28, 4.00 ± 0.70, and 15.7 ± 2.0 cells larva^-1^ (means ± SEs from triplicated experiments), respectively, and 8^th^ day, these densities had significantly increased to 10.9 ± 1.7, 35.2 ± 6.1, 57.6 ± 9.3, 23.4 ± 3.1, and 226 ± 31 cells larva^-1^ (means ± SEs from triplicated experiments), respectively, even though no symbiont sources were supplied after the 3^rd^ day (Fig 4, lines) (FP1: Δ deviance = 28.597, Δ df = 1, p < 0.001; FP2: Δ deviance = 33.199, Δ df = 1, p < 0.001; FP3: Δ deviance = 55.438, Δ df = 1, p < 0.001; C1: Δ deviance = 38.415, Δ df = 1, p < 0.001; C2: Δ deviance = 69.193, Δ df = 1, p < 0.001;). Consistent with the previous result, the highest density among the experimental groups inoculated with fecal pellets was found for FP3 on the 8^th^ day.

### Genetic compositions of Symbiodiniaceae within the fecal pellets and colonized larvae

**Fig 5:**
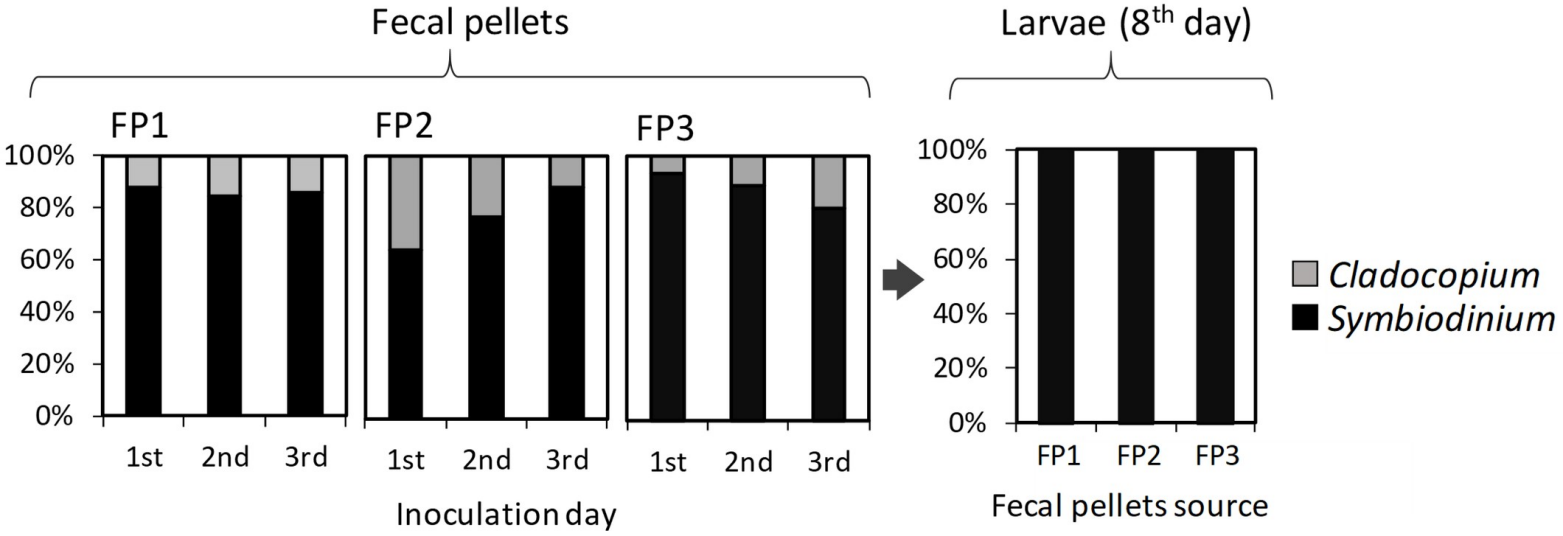
Genus compositions of Symbiodiniaceae in the fecal pellets provided on the three inoculation days and in the larvae on the 8^th^ day. The compositions in the pellets from each giant clam source were separately monitored by quantitative PCR. *Durusdinium* was not detected and is thus not shown in the legend.

Genus compositions of Symbiodiniaceae in the fecal pellets were monitored daily for each giant clam source by means of quantitative PCR and are shown in Fig 5. Across the total 3-days inoculation periods, the averaged compositions of *Symbiodinium* and *Cladocopium* in the fecal pellets from three giant clam individuals were 82.3 ± 5.2% and 17.7 ± 5.2%, respectively. More specifically, the percentages of *Symbiodinium* and *Cladocopium* in the fecal pellets from individuals 1, 2 and 3 were 85.9 ± 1.8% and 14.1 ± 1.8%, 76.1 ± 11.4% and 23.9 ± 11.4%, and 84.8 ± 10.1% and 15.2 ± 10.1%, respectively. Interestingly, the genus composition within the larvae retrieved on the 8^th^ day differed from that in the fecal pellets. Based on the quantitative PCR results, only *Symbiodinium* was detected in the larvae belonging to the experimental groups FP1, FP2, and FP3. Similar results were found in the larvae supplied the mixture of AJIS2-C2 (*S*. *microadriaticum*) and CCMP2556 (*D*. *trenchii*) (C1) and in those provided the mixture of TsIs-H4 (*S*. *tridacnidorum*) and TsIs-G10 (*Durusdinium* sp.) (C2). Even though these strains were supplied at equal cell densities, on the 8^th^ day, the percentages of *Symbiodinium* (AJIS2-C2 and TsIs-H4) were 93.8 ± 2.0% and 96.4 ± 1.6%, respectively, and these levels were higher than those of *Durusdinium* (CCMP2556 and TsIs-G10).

Although *Cladocopium* was not detected in the larvae by quantitative PCR methodology, Symbiodiniaceae types/species identification for the larvae in FP3 by DNA cloning revealed a minor occurrence of *C*. *goreaui* (1 read/total 116 readings of DNA clones), and *Symbiodinium* consisted of *S*. *tridacnidorum* (114 reads/116) and *S*. *natans* (1 read/116). *S*. *tridacnidorum* was recovered in all 49 readings from the supplied fecal pellets.

## Discussion

### Inoculation of coral larvae with Symbiodiniaceae in fecal pellets

Contrary to the majority of brooding corals, which maternally inherit symbiont cells (vertical transmission), more than 80% species of gamete-spawning corals must acquire their own symbionts from their surrounding environments (horizontal transmission) [7]. In this experiment, we found that *A*. *tenuis* larvae acquired symbiont cells derived from *T*. *crocea* fecal pellets and allowed their proliferation, and these fecal pellets might represent one of the symbiont sources in the environment.

Numerous studies have demonstrated the mechanisms through which coral larvae acquire Symbiodiniaceae. The low overlap between the genetic types of Symbiodiniaceae in the sediments of the Great Barrier Reef and those taken up by *A*. *tenuis* and *A*. *millepora* juveniles indicate that these corals might be capable of a degree of selectivity when acquiring symbionts [16]. In contrast, some studies have suggested that coral larvae might nonselectively acquire symbiont cells, but whether a stable symbiotic relationship is later successfully established or not could still be selective. Dunn & Weis [38] found that elimination of specific symbionts from *Fungia scutaria* larvae by a post-phagocytic response that induces apoptosis. Rejection of certain types of Symbiodiniaceae from coral larvae has also reported in *A*. *tenuis* [39, 40], which confirms a role for cell surface recognition molecules in controlling the postphagocytosis process [38]. In our current experiment, the colonization rate of larvae incubated with fecal pellets remained constant for at least five days even after termination of fecal pellet supply, and the symbiont cell densities per larva were significantly increased. These facts suggested the successful establishment of symbiosis and further growth of the acquired symbionts in the larvae, and those were free from host rejection even if the symbiont source was of noncoral origin and was subjected to the digestion process of giant clams.

Among the experimental groups incubated with fecal pellets, the larvae supplied pellets from individual 3 (FP3) showed the highest colonization rate and the highest cell density on the last day of the experiment. This successful establishment might be attributed to the high activity of the supplied cells. Morishima et al. [26] also reported that fecal pellets containing more active symbiont cells (*Fv/Fm* > 0.4) could induce increased establishment of symbiosis in *T*. *squamosa*. To the best of our knowledge, a possible mechanism responsible for a higher colonization rate coupled with a higher photosynthetic performance has not been reported, although more active symbionts might proliferate in larvae and contribute to host physiology. Previous studies have shown that coral juveniles harboring photosynthetically active Symbiodiniaceae that translocate more photosynthate to the hosts exhibit a growth advantage [41].

### Selection of Symbiodiniaceae species

Interestingly, the genus composition in the larvae retrieved on the 8^th^ day mostly consisted of the genus *Symbiodinium*, and the percentage was higher than that in the fecal pellets. In the control larvae that were supplied culture strains of *S*. *tridacnidorum* and *Durusdinium* sp. (C2), the symbiont mostly consisted of *Symbiodinium* (96.4 ± 1.4%) rather than *Durusdinium*, regardless of whether they were provided in equal densities. Similar but more obvious phenomena were found in the larvae belonging to the FP3 group that were retrieved on the 8^th^ day: only *Symbiodinium* was detected in these larvae by quantitative PCR, and DNA cloning identified the species as *S*. *tridacnidorum*, which exhibited significant domination in the population. Such selective acquisition and retention of specific Symbiodiniaceae in host cnidarians has been observed in *Acropora* corals [16] and the sea anemone *Exaiptasia pallida* [42]. Yamashita et al. [13] also found the selective acquisition and/or maintenance of specific Symbiodiniaceae genera in coral recruits kept in the natural environment. They analyzed a significant number of *Acropora* recruits (351 individuals) that had artificially or naturally settled in the environment and reported that only 8.8% of recruits harbored *Cladocopium* (formerly known as clade C), whereas 97.1% of recruits harbored *Symbiodinium* (formerly known as clade A) and/or *Durusdinium* (formerly known as clade D) despite the symbiodiniacean population in the environment being mainly composed of *Cladocopium*. The authors then observed the attraction behavior of several types/species of Symbiodiniaceae to *Acropora* larvae and compared the colonized ratio in the larvae [40]. Based on their results, some of the Symbiodiniaceae strains were obviously attracted to the larvae, and among them, specific types/species of *Symbiodinium* and *Durusdinium* were acquired by the larvae. These findings suggest that the initial establishment of coral–Symbiodiniaceae symbiosis is not random, and the infection mechanism appear to comprise two steps, namely an initial attraction step and subsequent selective uptake by the coral, even though we did not observe any attraction of Symbiodiniaceae cells in the fecal pellets. Although not the case of corals, *Symbiodinium* was dominantly found in the smaller individuals of the giant clam *Tridacna squamosa* [32]. Generally, it is believed that genus *Symbiodinium* is relatively insensitive to environmental stress, and often referred to as “weeds” among Symbiodiniaceae [13, 32, 43]. Such weedy nature might be suitable for the initial survival of coral larvae.

### Are fecal pellets of tridacnine clams feasible symbiont sources in the environment?

In our experiment, *S*. *tridacnidorum* showed the highest colonization rate and highest density in the larvae belonging to the C2 group, as well as in the larvae provided the fecal pellets. As insisted by its name, this species has been dominantly found in tridacnine clams [32, 44, 45]. It also occurs in association with a stinging hydroid and an upside-down jellyfish belonging to the genus *Cassiopea* [44]. Based on these findings, this species might not be native to scleractinian corals. At the same time, this species is acquired and maintainable in the larvae [40] and juveniles [46] of *Acropora tenuis*. Yuyama et al. [46] reported that *S*. *tridacnidorum* (a culture strain PL-TS-1, type A3) exhibited high density in juveniles and promoted juvenile growth. The coral *Seriatopora hystrix* is also known to possess *S*. *tridacnidorum*, which can be both vertically and horizontally transmitted [47].

The biogeography of host-free Symbiodiniaceae found within sediment of the Great Barrier Reef was intensively investigated [16], and *Symbiodinium* (described in their report as clade A) and *Cladocopium* (described as clade C) were found to be the major components in the sediments, which was consistent with previous reports of these genera occurring in high abundances in Pacific sediment communities [48, 49]. In particular to the specific offshore region of the Great Barrier Reef, *S*. *tridacnidorum* (described in their report as type A3) as well as *Cladocopium* (described as C15) and *Durusdinium* (described as D1) dominated in the sediment community [16]. Interestingly, rapid rates of colonization and proliferation of symbionts in coral juveniles exposed to these sediment treatments were recognized [16]. It was once reported by Littleman et al. [50] that on the Great Barrier Reef, symbiodiniacean densities were estimated to be surprisingly as high as approximately 1000–4000 cells ml^-1^ in the sediment samples, which was much higher than that in the water column (up to 80 cells ml^-1^), and these environmental populations are thought to influence the uptake of symbionts by both larvae and juvenile corals [51–53].

What are the origins of these host-free Symbiodiniaceae cells in these environmental pools? One of the candidates could be the population expelled from corals, which are known to maintain cell division [22] or competency in the photosystem [17, 18]. Similar to the cases in corals, the population expelled from tridacnine clams is photosynthetically active [23, 24, 26], and the cells in the fecal pellets expelled each day were reported to represent 3~6% of the total population in clam individuals [26]. Morishima et al. [26] estimated that at least 10^8^ cells of Symbiodiniaceae were hosted in even relatively small individuals of *T*. *crocea* (shell size = 62 mm-67.8 mm) and could be released to the environment; thus, 3~6 million cells might accumulate daily due to the contribution of a single tridacnine clam, which could be regarded as a significant source for host-free Symbiodiniaceae cells as well as those from corals. Because fecal pellets from giant clams present a negative buoyancy, they can transport Symbiodiniaceae more rapidly than that achieved with coral expulsion, which could contribute to the richness of symbiont pools on the sediment. If those populations from tridacnine clams are indeed able to colonize and proliferate even in corals, as indicated by this study, the rich inhabitation of tridacnine clams should also be regarded as a part of coral reef conservation. Furthermore, because the fecal pellet-derived symbionts are overflowed populations that suitably grew in tridacnine clams, the mechanism might increase the possibility for coral juveniles to access more “environmentally suitable” Symbiodiniaceae genera or species, which should be further investigated.

## Conclusion

The successful establishment of symbiosis and the further growth of Symbiodiniaceae acquired from fecal pellets of *Tridacna crocea* in *Acropora* larvae suggest an as-yet-unknown mechanism for the transport of symbionts to corals. Photosynthetically active Symbiodiniaceae cells in fecal pellets might induce higher acquisition and proliferation rates of the cells in the coral larvae. However, only a specific genus, *Symbiodinium*, in the pellets is taken up by the larvae, which confirms that coral larvae possess a selection mechanism, as has been suggested by many studies. Because of the pellets’ negative buoyancy, they can rapidly transport active Symbiodiniaceae to the coral reef sediment while avoiding dispersion of the cells, which might at least partly explain why rich Symbiodiniaceae pools are found in reef sediments.

## Supporting information

S1 Fig A diagonal rotator used for the inoculation experiment. (TIF)

S2 Fig Experimental scheme. (TIF)

S1 Data (XLSX)
