# Supplementary figures and images for: Fecal pellets of giant clams as a route for transporting Symbiodiniaceae to corals
Source: PLoS One. 2020 Dec 16;15(12):e0243087. doi: 10.1371/journal.pone.0243087 (PMC7743926; doi:10.1371/journal.pone.0243087)

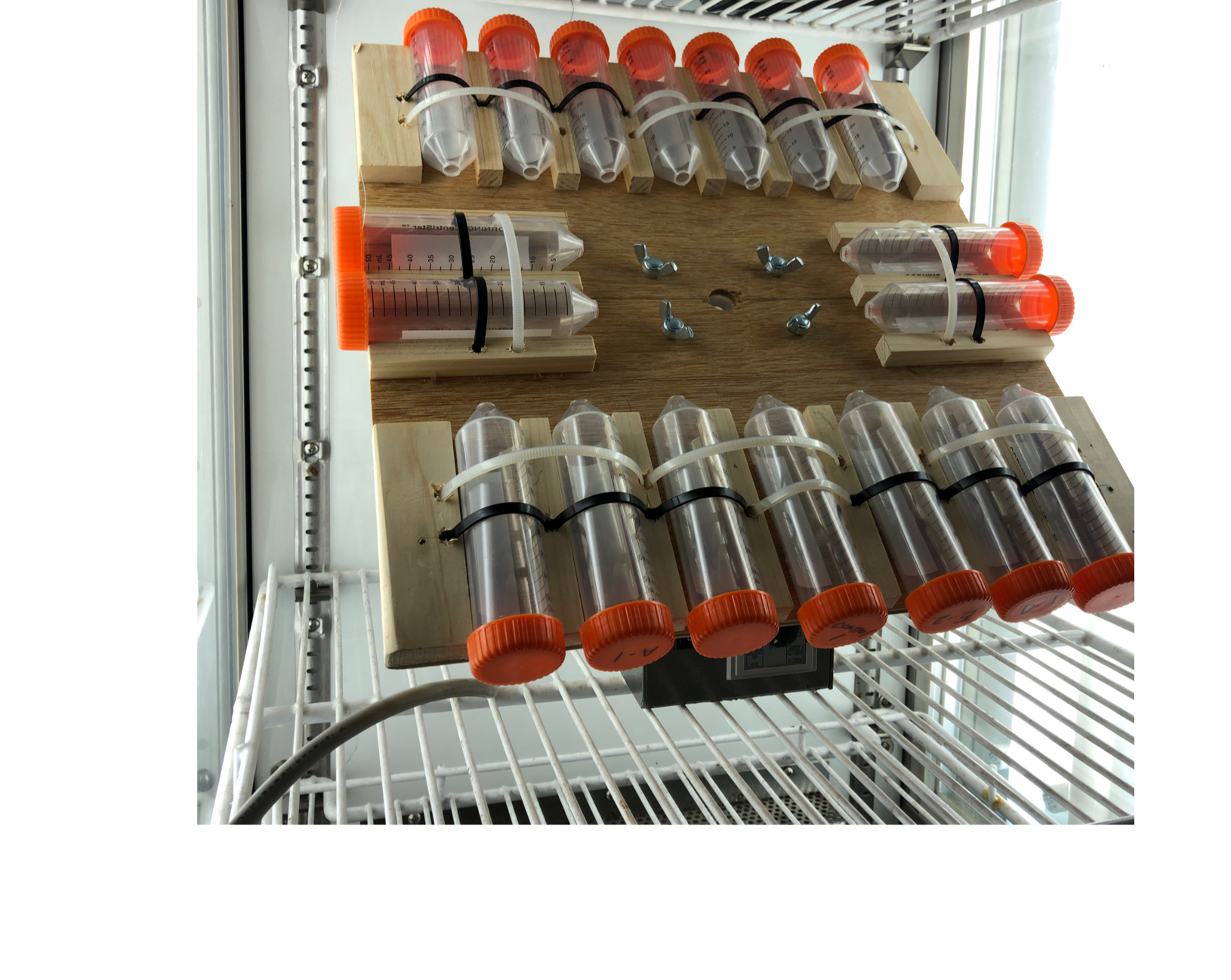

Supplement: S1 Fig — (TIF) [file pone.0243087.s001.tif]

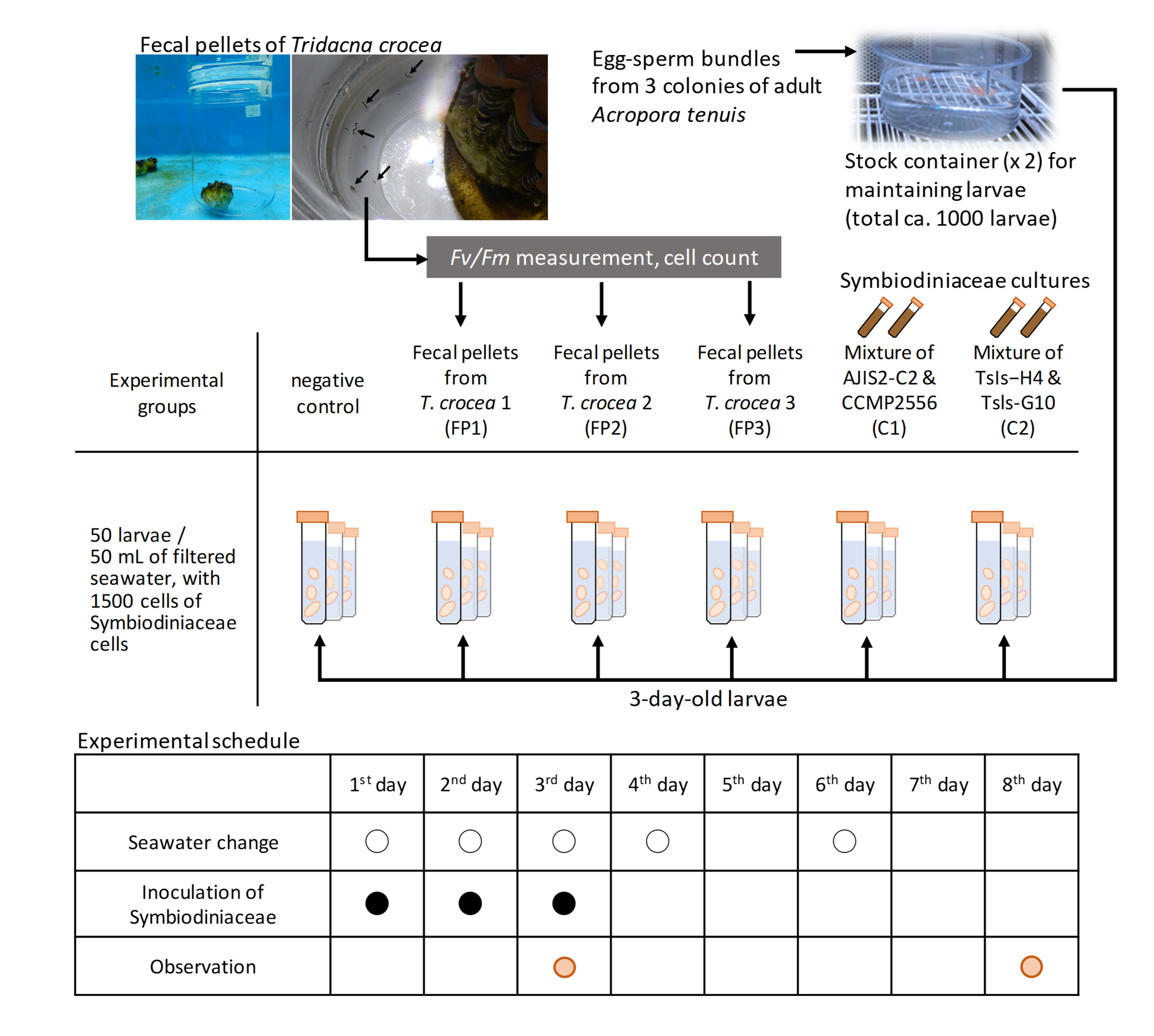

Supplement: S2 Fig — (TIF) [file pone.0243087.s002.tif]
